# Tick-transmitted infections in Transvaal: consider Rickettsia africae.

**DOI:** 10.3201/eid0501.990127

**Published:** 1999

**Authors:** P E Fournier, J Beytout, D Raoult


**M. Gagua,‡ Alexandre B. Predtechenski,§ Irina V. Tarasevich,† and Didier Raoult*†**
*Université de la Méditerranée, Marseille, France; †Russian Academy of Medical Sciences, Moscow, Russia; ‡Moscow Municipal Disinfection Center, Moscow, Russia; and §Research Center of Virology, Russia

### References

1. Raoult D, Roux V. Rickettsioses as paradigms of new or emerging infectious diseases. Clin Microbiol Rev 1997;10:694-719.
2. Maurin M, Raoult D. *Bartonella (Rochalimaea) quintana* infections. Clin Microbiol Rev 1996;9:273-92.
3. Johnson WD. Borrelia species (relapsing fever). In: Mandell GL, Douglas RG, Bennet JE, editors. Principles and practice of infectious diseases. 3rd ed. Edinburgh: Churchill Livingstone; 1990. p. 1816-8.
4. Patterson KD. Typhus and its control in Russia, 1870-1940. Med History 1993;37:361-81.
5. Raoult D, Ndihokubwayo JB, Tissot-Dupont H, Roux V, Faugere B, Abegbinni R, et al. Outbreak of epidemic typhus associated with trench fever in Burundi. Lancet 1998;352:353-8.
6. Tarasevich IV, Zemskaya AA, Dremova VP, Frolova AI, Hudobin VV, Lange AB. Human lice (diagnosis, medical significanse, methods of elimination) [in Russian]. Moscow: Medzdrav USSR; 1990. p. 5-7.
7. Tarasevich IV, Fetisova NF. Classical typhus [in Russian]. ZniSO (Health of Population and Environment) 1995;2:9-13.
8. Raoult D, Roux V, Ndihokubwayo JB, Bise G, Baudon D, Martet G, et al. Jail fever (epidemic typhus) outbreak in Burundi. Emerg Inf Dis 1997;3:357-60.
9. Eremeeva ME, Balayeva NM, Raoult D. Serological response of patients suffering from primary and recrudescent typhus: comparison of complement fixation reaction, Weil-Felix test, microimmunofluorescence, and immunoblotting. Clin Diagn Lab Immunol 1994;1:318-24.
10. Tarasevich IV, Rydkina E, Raoult D. An outbreak of epidemic typhus in Russia. Lancet 1998;352:1151.
11. Brouqui P, Houpikian P, Tissot Dupont H, Toubiana P, Obadia Y, Lafay V, et al. Survey of the seroprevalence of *Bartonella quintana* in homeless people. Clin Infect Dis 1996;23:756-9.
12. Comer JA, Flynn C, Regnery RL, Vlahov D, Childs JE. Antibodies to Bartonella species in inner-city intravenous drug users in Baltimore, MD. Arch Intern Med 1996;156:2491-5.
13. Drancourt M, Mainardi JL, Brouqui P, Vandenesch F, Carta A, Lehnert F, et al. *Bartonella (Rochalimaea) quintana* endocarditis in three homeless men. N Engl J Med 1995;332:419-23.
14. Jackson LA, Spach DH, Kippen DA, Sugg NK, Regnery RL, Sayers MH, et al. Seroprevalence to *Bartonella quintana* among patients at a community clinic in downtown Seattle. J Infect Dis 1996;173:1023-6.
15. Koehler JE, Sanchez MA, Garrido CS, Whitfeld MJ, Chen FM, Berger TG, et al. Molecular epidemiology of bartonella infections in patients with bacillary angiomatosis-peliosis. N Engl J Med 1997;337:1876-83.
16. Raoult D, Drancourt M, Carta A, Gastaut JA. *Bartonella (Rochalimaea) quintana* isolation in patient with chronic adenopathy, lymphopenia, and a cat. Lancet 1994;343:977.
17. Gromashevski LB, Vaindrach GM. Relapsing typhus [in Russian]. Moscow: Medgiz; 1946. p. 78-96.
18. Kim KC, Ludwig HW. The family classification of the Anoplura. Systematic Entomology 1978;3:249-84.
19. Regnery RL, Spruill CL, Plikaytis BD. Genotypic identification of rickettsiae and estimation of intraspecies sequence divergence for portions of two rickettsial genes. J Bacteriol 1991;173:1576-89.
20. Roux V, Rydkina E, Eremeeva M, Raoult D. Citrate synthase gene comparison, a new tool for phylogenetic analysis, and its application for the Rickettsiae. Int J Syst Bacteriol 1997;47:252-61.
21. Roux V, Raoult D. The 16S-23S rRNA intergenic spacer region of Bartonella (Rochalimaea) species is longer than usually described in other bacteria. Gene 1995;156:107-11.
22. Birtles RJ, Raoult D. Comparison of partial citrate synthase gene (gltA) sequences for phylogenetic analysis of Bartonella species. Int J Syst Bacteriol 1996;46:891-7.
23. Mitko E. To count homeless in autumn [in Russian]. Vechernyaya Moskva (Evening Moscow) Newspaper 1997; 137.
24. Raoult D, Fournier PE, Drancourt M, Marrie TJ, Etienne J, Cosserat J, et al. Diagnosis of 22 new cases of Bartonella endocarditis. Ann Intern Med 1996;125:646-52.
25. Parrott JH, Dure L, Sullender W, Buraphacheep W, Frye TA, Galliani CA, et al. Central nervous system infection associated with *Bartonella quintana*: a report of two cases. Pediatrics 1997;100:403-8.
26. Case Records of the Massachusetts General Hospital. N Engl J Med 1998;338:112-9.
27. Spach DH, Kanter AS, Dougherty MJ, Larson AM, Coyle MB, Brenner DJ, et al. *Bartonella (Rochalimaea) quintana* bacteremia in inner-city patients with chronic alcoholism. N Engl J Med 1995;332:424-8.
28. Stein A, Raoult D. Return of trench fever. Lancet 1995;345:450-1.
29. Jackson LA, Spach DH. Emergence of *Bartonella quintana* infection among homeless persons. Emerg Infect Dis 1996;2:141-4.
30. Relman DA. Has trench fever returned? N Engl J Med 1995;332:463-4.
31. Walker DH, Barbour AG, Oliver JH, Lane RS, Dumler JS, Dennis DT, et al. Emerging bacterial zoonotic and vector-borne diseases. Ecological and epidemiological factors. JAMA 1996;275:463-9.

### Tick-Transmitted Infections in Transvaal: Consider *Rickettsia africae*

**To the Editor:** We report a case of African tick-bite fever (ATBF) in a 54-year-old French hunter returning to France on 21 April 1997, after a 15-day visit to Transvaal, South Africa. While traveling in the veld, the hunter removed (but did not keep) two ticks from his left leg. Two days later, he observed eschars at the bite sites. Within 5 days, he had high fever (39.5°C) and headache and decided to fly back to France, where he was admitted to the Infectious Diseases Department in the Hotel Dieu Hospital in Clermont-Ferrand. The patient's clinical symptoms were persistent fever, severe headache, and two inflammatory eschars on the left leg. Laboratory results were normal. On 22 April, an acute-phase serum sample and eschar biopsy were sent to our laboratory. The patient was treated with 200 mg per day doxycycline for 10 days. His symptoms resolved. A second serum sample was collected on 13 May.

Microimmunofluorescence was performed as previously described (1). Although the acute-phase serologic results were negative, the convalescent-phase serum exhibited anti–*R. africae* and anti–*R. conorii* titers of 16 for immunoglobulin (Ig) G and 8 for IgM. Sera were adsorbed with *R. conorii* and *R. africae* antigens (2), and serologic testing and Western blot analysis (1) were performed on the resultant supernatants. Cross-adsorption of the convalescent-phase serum caused the homologous and heterologous antibodies to disappear when adsorption was performed with *R. africae* antigens; only homologous antibodies disappeared when adsorption was performed with *R. conorii*. Western immunoblot, performed with the same adsorbed serum, indicated *R. africae* infection by demonstrating a specific reactivity pattern with *R. africae*–specific antigens in the 110-kDa to 145-kDa region (2). An inoculation eschar biopsy specimen was injected into human embryonic lung fibroblasts, according to the centrifugation shell-vial technique (3). After 6 days' incubation at 32°C, a Gimenez staining of methanol-fixed human embryonic lung fibroblasts showed rickettsialike bacilli. The strain was identified by direct immunofluorescence performed on the cells with an anti–*R. africae* monoclonal antibody (4). Moreover, DNA was extracted from the ground eschar biopsy specimen and from 200 µL of shell-vial supernatant, by using a QIAmp Tissue kit (QIAGEN GmbH, Hilden, Germany), according to the manufacturer's instructions. These extracts were used as templates with primers complementary to a portion of the coding sequence of the rOmpA encoding gene in a polymerase chain reaction (PCR) assay (5), and the base sequences of the resulting PCR products were determined (5). The sequence obtained by both methods was the same as the *R. africae* sequence in Genbank (100% similarity).

Since first described in Africa in 1910, tick-transmitted rickettsioses have been imputed to a single rickettsial species, *Rickettsia conorii*, although two distinct clinical illnesses have been observed (6): an urban form in patients in contact with dogs and their ticks (*Rhipicephalus* spp.) characterized by fever, headache, myalgia, cutaneous rash, and a lesion at the site of the tick bite (7), and a rural form in patients in contact with cattle or game and their ticks (*Amblyomma* spp.) characterized by mild signs and frequent lack of rash (8).

Although *R. africae* was initially isolated from *Amblyomma* cattle ticks in 1973, the first evidence of its pathogenic role in humans was seen in 1992 in a patient who, after a tick bite, had fever, an inoculation eschar, regional lymphadenopathy, but no cutaneous rash (9). Since then, an additional 20 cases of *R. africae*–related infections have been reported in travelers returning from Zimbabwe and South Africa (2,10).

*R. conorii* has long been considered the only African spotted fever group rickettsia, responsible for both Mediterranean spotted fever and ATBF. Since the first case was described (9), most of the 20 reported cases of ATBF occurred as outbreaks (2,10) in Europeans returning from Zimbabwe and South Africa. The occurrence of concomitant ATBF cases is unusual since Mediterranean spotted fever is generally sporadic and is likely related to the biologic characteristics of the recognized vector of *R. africae*, *Amblyomma* spp. ticks. While both are nonnidicolous ticks, *Amblyomma* spp. and *Rhipicephalus* spp. exhibit very different host-seeking behavior (11). *Amblyomma* spp. are ticks of cattle and wild ungulates, are not host-specific, and can readily feed on humans; they are "hunter ticks" and exhibit an "attack strategy" (in response to stimuli they specifically converge on nearby hosts). *Rhipicephalus* spp. are dog ticks and vectors of *R. conorii*; very host-specific, they exhibit an "ambush strategy" (they are passive and remain quiescent in their habitat until a vertebrate host passes). Up to 72% of *A. hebraeum* are infected with *Rickettsia*-like organisms, in particular *R. africae* (12); *Amblyomma* spp. are widely distributed in rural areas in sub-Saharan Africa (13) and prevalence of *A. hebraeum* ticks, incidence of ATBF cases, and prevalence of *R. africae* antibodies have been strongly linked (14). Rural Africans are also commonly infected with *R. africae*, usually at a young age (14). In Zimbabwe, Kelly et al. (15) demonstrated that 55% of the tested human sera had antibodies against *R. africae*.

ATBF usually has specific clinical features: shorter incubation period than for Mediterranean spotted fever, multiple inoculation eschars (related to the host-seeking behavior and host-specificity of *Amblyomma* spp. ticks, which are "attack ticks" [15]), regional lymphadenopathies, frequent lack of cutaneous rash or a pale vesicular eruption, and absence of complications (2). Although only 22 proven cases have been described so far (including the present case), ATBF has been recognized as a commonly encountered disease in southern Africa since 1900 (8,16). Epidemiologic and clinical features indicate that several cases previously diagnosed on the basis of serology results only as *R. conorii*-caused may have been caused by *R. africae*.

Given the serologic cross-reactivity among spotted fever group rickettsiae, microimmunofluorescence, the easiest serologic method, may not be sufficient for the etiologic diagnosis of a rickettsial spotted fever. A definitive diagnosis of ATBF requires either additional serologic procedures, such as cross-adsorption or Western blot, or the use of PCR or culture. As for PCR, rOmpA-amplification possesses sufficient sequence heterogeneity among the spotted fever group rickettsiae to be used as an identification tool (5). The centrifugation-shell vial-cell culture (3), used routinely in our laboratory, reliably isolates strictly intracellular bacteria, including rickettsia, from blood and tissue specimens, especially eschar biopsies (the specimen of choice for isolation procedures or genomic detection). We noted cross-reactions between *R. africae* and *R. conorii.* Cross-adsorption between anti–*R. africae* and anti–*R. conorii* antibodies and Western blots confirmed that the antibodies we detected were directed specifically at *R. africae*. Furthermore, both PCR and cell culture confirmed the diagnosis of *R. africae* infection.

ATBF appears to be an important emerging disease in visitors to rural areas of southern Africa. *R. africae* should be considered a potential pathogen in patients returning from such areas who have fever, headache, multiple inoculation eschars, or regional lymphadenopathy after a tick bite.

**Pierre-Edouard Fournier,* Jean Beytout,† and Didier Raoult***
*Université de la Méditerranée, Marseille, France; and †Centre Hospitalier Régional Hotel Dieu, Clermont-Ferrand, France

### References

1. Teysseire N, Raoult D. Comparison of Western immunoblotting and microimmunofluoresence for diagnosis of Mediterranean spotted fever. J Clin Microbiol 1992;30:455-60.
2. Brouqui P, Harle JR, Delmont J, Frances C, Weiller PJ, Raoult D. African tick bite fever: an imported spotless rickettsiosis. Arch Int Med 1997;157:119-24.
3. Marrero M, Raoult D. Centrifugation-shell vial technique for rapid detection of Mediterranean spotted fever rickettsia in blood culture. Am J Trop Med Hyg 1989;40:197-9.
4. Xu W, Beati L, Raoult D. Characterization of and application of monoclonal antibodies against *Rickettsia africae*, a newly recognized species of spotted fever group rickettsia. J Clin Microbiol 1997;35:64-70.
5. Roux V, Fournier PE, Raoult D. Differentiation of spotted fever group rickettsiae by sequencing and analysis of restriction fragment length polymorphism of PCR amplified DNA of the gene encoding the protein rOmpA. J Clin Microbiol 1996;34:2058-65.
6. Conor A, Bruch A. Une fièvre éruptive observée en Tunisie. Bull Soc Pathol Exot Filial 1910;8:492-6.
7. Sant'Anna JF. On a disease in man following tick-bites and occurring in Lourenço Marques. Parasitology 1912;4:87-8.
8. Troup JM, Pijper A. Tick-bite fever in Southern Africa. Lancet 1938;ii:1183-6.
9. Kelly P, Matthewman LA, Beati L, Raoult D, Mason P, Dreary M, et al. African tick-bite fever—a new spotted fever group rickettsiosis under an old name. Lancet 1992;340:982-3.
10. Fournier PE, Roux V, Caumes E, Donzel M, Raoult D. An outbreak of *Rickettsia africae* infections among participants in an adventure race from South Africa. Clin Infect Dis. In press 1998.
11. Sonenshine DE. Ecology of non-nidicolous ticks. In: Sonenshine DE, editor. Biology of ticks. Oxford (NY): Oxford University Press; 1993. p. 3-65.
12. Beati L, Kelly PJ, Matthewman LA, Mason P, Raoult D. Prevalence of Rickettsia-like organisms and spotted fever group Rickettsiae in ticks (Acari: *Ixodidae*) from Zimbabwe. J Med Entomol 1995;32:787-92.
13. Kelly PJ, Beati L, Mason PR, Matthewman LA, Roux V, Raoult D. *Rickettsia africae* sp nov, the etiological agent of African tick bite fever. Int J Syst Bacteriol 1996;46:611-4.
14. Tissot-Dupont H, Brouqui P, Faugere B, Raoult D. Prevalence of antibodies to *Coxiella burnetii, Rickettsia conorii*, and *Rickettsia typhi* in seven African countries. Clin Infect Dis 1995;21:1126-33.
15. Kelly PJ, Mason PR, Matthewman LA, Raoult D. Seroepidemiology of spotted fever group rickettsial infections in human in Zimbabwe. Am J Trop Med Hyg 1991;94:304-9.

16. Gear JHS, Bevan C. An outbreak of tick-bite fever. S Afr Med J 1936;10:485-8.

### Extended-Spectrum Beta-Lactamase-Producing *Salmonella* Enteritidis in Trinidad and Tobago

**To the Editor:** *Salmonella* Enteritidis, a predominantly localized pathogen of the human gastrointestinal tract, can become invasive in very young, very old, malnourished, and immunocompromised patients. In recent years, *S.* Enteritidis has emerged as a major intestinal pathogen in Trinidad and Tobago (population 1.2 million); in 1997, *S.* Enteritidis caused 79 (66%) of 119 culture-confirmed salmonella infections, in contrast to 18 (18%) of 99, 48 (47%) of 102, and 107 (61%) of 178 in 1994, 1995, and 1996, respectively. Increased incidence of *S.* Enteritidis infections has been reported worldwide (1,2). Of 216 human *S.* Enteritidis isolates tested for antimicrobial susceptibility between 1994 and 1996 in Trinidad, none were resistant to cephalosporins, aminoglycosides, ampicillin, trimethoprim-sulphamethoxazole, chloramphenicol, and norfloxacin/ciprofloxacin by the Kirby-Bauer disk diffusion method, which uses the National Committee for Clinical Laboratory Standards (NCCLS) breakpoints (3).

Here we report an unusual isolate of *S.* Enteritidis resistant to all penicillins and cephalosporins—including third-generation cephalosporins, gentamicin, tobramicin, and trimethoprim-sulphamethoxazole—by the Kirby-Bauer disk diffusion method. Amoxicillin-clavulanate and piperacillin-tazobactam disks gave zone sizes of 15 mm and 19 mm, respectively, which are classified as intermediate in the NCCLS guidelines. This isolate was recovered from the blood culture of a febrile, nonneutropenic patient with multiple myeloma on two occasions 24 hours apart in March 1998. The isolate was sensitive only to ofloxacin and imipenem. Admitted to the hospital with compressed fracture of the spine for physiotherapy in December 1997, the patient had several febrile episodes and received several courses of multiple empirically prescribed antibiotics (cefotaxime, gentamicin, and piperacillin). The patient had not traveled abroad during the previous 6 months.

Because cephalosporin resistance in salmonellae has not been reported before in the Caribbean, we investigated the mechanism behind this third-generation cephalosporin resistance further. Using amoxicillin-clavulanate in combination with ceftazidime, ceftriaxone, and aztreonam, we performed the double disk synergy test to determine whether this strain was an extended-spectrum beta-lactamase producer as described elsewhere (3); augmentation of the zone at the junction of amoxicillin-clavulanate and aztreonam/ceftriaxone/ceftazidime zones confirmed that indeed it was.

In the past few years, third-generation cephalosporin resistance in *S.* Enteritidis has been described in Europe (4), the United States (5), Turkey (6), India (7,8), and Argentina (9). Few reports exist of extended-spectrum beta-lactamase–mediated third-generation cephalosporin resistance in *Salmonella* spp. To our knowledge, this is the first report of this type of resistance among *S.* Enteritidis in the Caribbean. This patient was treated with ciprofloxacin for 1 week; subsequent blood cultures were negative.

This unusual isolate highlights the need to establish an antimicrobial resistance surveillance network for Salmonella isolates, including *S.* Enteritidis, to monitor the trends and new types of resistance mechanisms in the Caribbean. An epidemiologic study of *S.* Enteritidis infections is being planned to describe the extent of the problem and to define risk factors and vehicles of human infections in three Caribbean countries, including Trinidad and Tobago.

**B.P. Cherian,* Nicole Singh,* W. Charles,* and P. Prabhakar*†**

*Port of Spain General Hospital, Port of Spain, Trinidad; and †Caribbean Epidemiology Center (CAREC), Port of Spain, Trinidad

### References

1. Centers for Disease Control and Prevention. Salmonella surveillance annual tabulation summary, 1993-1995. Atlanta: U.S. Department of Health and Human Services; 1997.
2. Communicable Disease Surveillance Center. Salmonella in humans: PHLS salmonella data set, England and Wales, 1981-1996. London: The Center; 1997.
3. National Committee for Clinical Laboratory Standards. Performance standards for the anti-microbial disk susceptibility tests for bacteria that grow aerobically. Approved standard M7 - A4. Villanova (PA): The Committee; 1997.

